# A labelled dataset to classify direct deforestation drivers from Earth Observation imagery in Cameroon

**DOI:** 10.1038/s41597-024-03384-z

**Published:** 2024-05-31

**Authors:** Amandine Debus, Emilie Beauchamp, James Acworth, Achille Ewolo, Justin Kamga, Astrid Verhegghen, Christiane Zébazé, Emily R. Lines

**Affiliations:** 1 Department of Geography, University of Cambridge, Downing Place, Cambridge, CB2 3EN United Kingdom; 2 International Institute for Sustainable Development (IISD), 111 Lombard Avenue, Suite 325, Winnipeg, Manitoba R3B 0T4 Canada; 3 United Nations Development Programme (UNDP), Nouvelle route Bastos B.P. 836, Yaoundé, Cameroun; 4 Centre for Environment and Development (CED), Etoa-Meki, Yaoundé, P.O Box 3430, Cameroon; 5 Forêts et Développement Rural (FODER), Derrière Usine Bastos, Rue 228, 11417 Yaoundé, Cameroon; 6 European Commission, Joint Research Centre (JRC), Ispra, Italy; 7 ARHS Developments Italia S.R.L., Via Gabba Frattelli 1/A, 20121 Milan, Italy

**Keywords:** Environmental sciences, Forestry

## Abstract

Understanding direct deforestation drivers at a fine spatial and temporal scale is needed to design appropriate measures for forest management and monitoring. To achieve this, reference datasets with which to design Artificial Intelligence (AI) approaches to classify direct deforestation drivers within areas experiencing forest loss in a detailed, comprehensive and locally-adapted way are needed. This is the case for Cameroon, in the Congo Basin, which has known increasing deforestation rates in recent years. Here, we created an Earth Observation dataset with associated labels to classify detailed direct deforestation drivers in Cameroon, which includes satellite imagery (Landsat and PlanetScope) and auxiliary data on infrastructure and biophysical properties. The dataset provides the following fifteen labels: oil palm, timber, fruit, rubber and other-large scale plantations; grassland/shrubland; small-scale oil palm or maize plantations and other small-scale agriculture; mining; selective logging; infrastructure; wildfires; hunting; and other.

## Background & Summary

Africa, which is home to the second largest rainforest in the world, the Congo Basin^1^, experienced the largest continental annual forest area net change for 2010–2020^2^. Within the Congo Basin, Cameroon had the sharpest average annual rise in primary forest and tree cover loss between 2016 and 2021^3^, highlighting the importance of understanding drivers of Cameroonian deforestation and degradation dynamics, in order to make informed decisions to limit impacts^4^. Direct deforestation drivers describe the activities directly resulting in the land-use change (e.g. conversion to agricultural land, development of infrastructure)^1^. They are, however, difficult to track on the ground, and Earth Observation (EO) offers opportunities to cover large areas which can be difficult to access.

Attempts have been made to classify direct tropical deforestation and degradation drivers, and follow up land uses, in Indonesia^5^, Ethiopia^6^, Suriname^7^, the Republic of Congo^7^, the Democratic Republic of Congo^7^, and Africa as a whole^8,9^. These highlighted the potential of EO. However, they also emphasised the need for tailored and locally trained algorithms to make sure the decisions made to define the land use classes correspond to national deforestation dynamics. Global approaches also do not account for spatio-temporal heterogeneity in land use between countries, and usually lack diversity in the representation of classes^6,8^. In addition, detailed information about the drivers, i.e. beyond broad classes such as ‘agriculture’, is needed to monitor and target efforts towards specific drivers, but is usually lacking^6,8^. A common limitation cited for the development of such approaches across countries is the lack of reference datasets^10^. This was a limitation we faced when trying to design a detailed and comprehensive approach to automatically classify direct deforestation drivers in Cameroon. As a result, we created a new labelled dataset for Cameroon, presented here, collated and standardised from nine existing datasets. Our efforts show that the common trope that there is ‘not enough’ labelled data for spatial Artificial Intelligence (AI) applications for sub-Saharan Africa^11^ can be a misconception. Instead, we found that issues arise in finding and accessing open, geolocated and spatially explicit data in a uniform format, on a single platform, which is easy to use and download.

## Methods

### Overview

In order to create a new labelled EO dataset for deforestation drivers in Cameroon we followed these steps:

1. Identify appropriate drivers using key informant discussions and literature.
2. Convert the Global Forest Change (GFC) product to a shapefile format for the area covering Cameroon.
3. Identify suitable georeferenced data of land use in Cameroon.
4. Overlay these with GFC data to identify areas that had been recently deforested and extract forest loss masks.
5. Extract corresponding Landsat-8 single date images and NICFI PlanetScope monthly composites centred on the forest loss masks, and cross-reference these to driver data.
6. Extract corresponding auxiliary data.

**Fig. 1:**
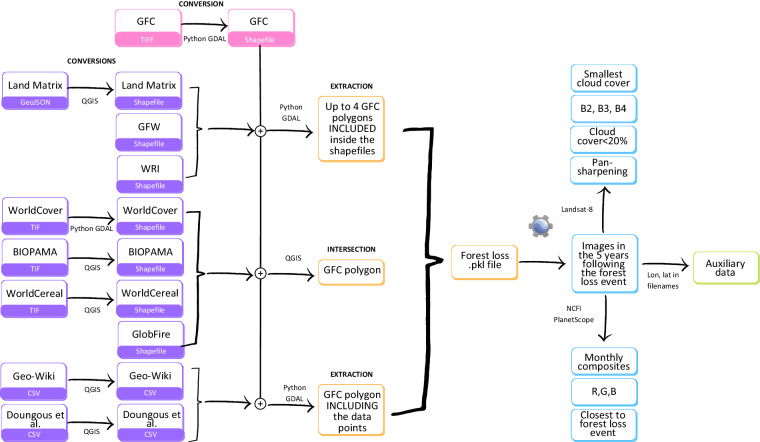
Workflow to generate a reference labelled dataset. In pink: the operation on the GFC product. In purple: operations on the data sources. In yellow: creation of forest loss pickle files. In blue: extraction of satellite imagery. In green: extraction of auxiliary data.

Resultant data are presented in formats useful for ML/AI applications - as sets of EO and auxiliary data. We present the EO and auxiliary data in three formats, according to large-scale (‘grouped’) deforestation drivers, according to fine-scale (‘detailed’) deforestation drivers, and as a time-series of data for ‘detailed’ drivers, and the labels separately for easy cross-reference. For each image, the label applies to the area delimited by the forest loss masks. The workflow is summarised on Fig. 1. Our dataset is freely available from Zenodo^12^.

### Defining the classification scheme

In order to define appropriate classes of deforestation drivers for our dataset, we reviewed academic and grey literature^13–23^, and carried out key-informant discussions with different experts in NGOs, academia, research institutes, intergovernmental organisations, and partnership initiatives. We further refined our classes based on the availability of data, and the final labels we used are explained in Table 1. Our classification includes drivers of degradation alongside deforestation, since degradation is often the first step before deforestation^24,25^ and impacts forest structure and function^26^.

**Table 1:** Degradation and deforestation drivers used to label the data and number of images downloaded for each driver. Each image is associated with one driver.

Grouped drivers^5^	Detailed drivers	Number of Landsat-8 images downloaded	Number of NICFI PlanetScope images downloaded
Plantation (large-scale)	Oil palm plantation	192	212
	Timber plantation	493	514
	Fruit plantation (e.g. banana)	63	63
	Rubber plantation	135	214
	Other large-scale plantation (e.g. tea, sugarcane)	127	130
Grassland/Shrubland	Grassland/Shrubland	97	100
Smallholder agriculture	Small-scale oil palm plantation	271	302
	Small-scale maize plantation	385	402
	Other small-scale agriculture	147	154
Other	Mining	215	272
	Selective logging	546	571
	Infrastructure	53	59
	Wildfire	152	170
	Hunting	132	139
	Other	100	100

### Classification of forest loss areas

We downloaded Global Forest Change (GFC)^27^ granules (https://storage.googleapis.com/earthenginepartners-hansen/GFC-2020-v1.8/download.html) for Cameroon (coordinates 0–10 N, 0–10E; 0–10 N, 10–20E; 10–20 N, 10–20E) for each year between 2015 and 2020. GFC corresponds to annual tree cover loss maps derived from Landsat data, with a 30-m resolution. We chose 2015–2020 as our period of study to make sure we use the most accurate and recent methodology^28^ used to derive GFC, to focus on recent deforestation patterns, and to best reflect the availability of the datasets used for labelling (more details in the section below).

We converted the GFC TIFF files to shapefiles. First, for each year, we binarised TIFF images with tree cover loss locations in the corresponding year. Then, we used the Python GDAL library to select the loss pixels and create one GFC shapefile per year. Each GFC shapefiles contains multiple shapes corresponding to tree loss areas in the given year. The tree cover loss from GFC is considered as a forest loss for this study.

### Selection of data for associated direct deforestation drivers

We used data from nine sources on land cover/land use in Cameroon:

- Shapefiles from Global Forest Watch (GFW) identifying areas of selective logging annually for 2015–2018^29^ (https://data.globalforestwatch.org/datasets/f5f24ef23b86444699aff1e23a0eb27b_3/explore?location=3.863682%2C12.477300%2C7.54).
- GeoJSON files from Land Matrix identifying fruit plantations present for the entire period 2018–2020, and timber plantations present for 2015–2020^30^, converted to shapefiles using QGIS (https://landmatrix.org/map/, by selecting ‘Cameroon’ in the ‘Country’ section).
- Shapefiles, selected individually from yearly GIS Cameroon data, from the World Resources Institute (WRI) Cameroon Forest Atlas identifying rubber plantations, oil palm plantations, fruit plantations, other large-scale plantations for 2015–2017 and 2019–2020, mining for 2019–2020^16^, as well as specific oil palm and rubber plantations (BioPalm, SocaPalm, SudCam, Hevecam) present for the entire period 2015–2020^16,23^ (https://data.globalforestwatch.org/search?layout=grid&q=cameroon%20GIS%20data&sort=Date%20Created%7Ccreated%7Cdesc), and hunting for 2015–2020^16^ (https://data.globalforestwatch.org/datasets/050ee10e13ec471ba9e2305f82759541_88/explore?location=5.368277%2C14.094700%2C6.74).
- TIF files from the European Space Agency (ESA) WorldCover map, identifying grassland/shrubland and permanent water bodies (for the ‘other’ class) for 2020^31^, converted to shapefiles using the Python GDAL library (https://viewer.esa-worldcover.org/worldcover/?language=en&bbox=-91.91076222321404,-57.011563963962864,117.24202780840146,33.059940763899064&overlay=false&bgLayer=OSM&date=2024-05-01&layer=WORLDCOVER_2020_MAP, by selecting ‘Administrative borders’>’Countries’ and clicking on Cameroon in ‘Download’>’Extent’).
- A CSV file from Geo-Wiki identifying the coordinates of wildfires, infrastructure and other small-scale plantations (cassava) in 2020^32,33^ (https://pure.iiasa.ac.at/id/eprint/17539/).
- A CSV file from Doungous *et al*. identifying the coordinates of other small-scale plantations (maize) in 2020^34^ (https://view.officeapps.live.com/op/view.aspx?src=https%3A%2F%2Fars.els-cdn.com%2Fcontent%2Fimage%2F1-s2.0-S0261219422001132-mmc1.xlsx&wdOrigin=BROWSELINK).
- A TIF file from BIOPAMA identifying small-scale oil palm plantation in 2019^35,36^, with the relevant layer extracted and converted to a shapefile on QGIS (https://zenodo.org/records/4473715).
- TIF files from WorldCereal identifying small-scale maize plantations and other small-scale plantations (wheat, barley, rye) in 2020^31^, converted to shapefiles on QGIS (https://zenodo.org/records/5571936).
- Shapefile from GlobFire identifying wildfires annually for 2015–2020^37,38^ (https://doi.pangaea.de/10.1594/PANGAEA.895835).

We manually checked for any duplicated information between different sources to remove them. If the data sources contradicted themselves, the data was also removed.

The data sources above identify land cover/land use at the time of survey, but do not provide information about whether deforestation or any other type of land use change happened there. We therefore overlaid each land use file with GFC annual shapefiles for each year where the land use has been identified. For each shapefile from GFW, Land Matrix, and WRI we looked for a GFC forest loss polygon contained in that shape and extracted that polygon as a shapefile and a corresponding pickle file (Python-specific format to serialise an object, i.e. to convert it to a linear form). For WorldCover, WorldCereal, BIOPAMA, and GlobFire, we intersected the shapefiles with the GFC shapefiles directly on QGIS and then extracted the intersections as individual forest loss polygons shapefiles and corresponding pickle files using the Python GDAL library. For WorldCover, we limited the number of forest loss shapefiles to the first hundred per year, following the ordered indices of the shapes in the intersection shapefile obtained with QGIS. For Geo-Wiki and Doungous *et al*. data, we extracted the coordinates of the datapoints in the CSV files, checked that they were included in Cameroon and looked for GFC polygons containing these coordinates to then extract these GFC forest loss polygons as shapefiles and generate the corresponding pickle files with the Python GDAL library. For WorldCover, WorldCereal, and Geo-Wiki, different shapefiles were created for each type of driver.

For large-scale plantations except fruit plantations, we extracted two additional GFC forest loss polygons from each shape in WRI shapefiles. For mining and fruit plantations, we extracted three additional GFC forest loss polygons from WRI shapefiles. This step was needed to obtain enough training data for these classes.

The steps above give us forest loss pickle files, or forest loss masks, where we know the deforestation drivers thanks to the data sources used. In other words, we obtain labels for our images, which are centred on the forest loss masks. The label will apply only to the area covered by the forest loss mask. The process to download the images is described in more detail in the section below. The labels are determined using the names of the data sources overlaid with the GFC shapefiles (e.g. ‘BioPalm.shp’ for BioPalm data corresponds to ‘Oil palm plantation’). In the case of data sources with multiple types of land uses, i.e. for agro-industrial plantations in WRI data, the label is given based on a manual inspection of each shape within the shapefiles and the use of additional data from the Cameroon Forest Atlas website^16^, giving us information about the crop type for each individually indexed shape.

### Images

We downloaded Landsat-8 and NICFI PlanetScope imagery from Google Earth Engine, using the multiprocessing package to enable parallel processing and therefore a faster download of images.

We downloaded images with the centroid of the forest loss polygons as the centre of the images. For Landsat-8 images, we selected the calibrated top-of-atmosphere (TOA) reflectance image, and pan-sharpened the 30-m resolution images to a 15 m resolution using the panchromatic band 8. For each forest loss polygon, we selected the image with the lowest cloud clover available in the five years following the forest loss event (which corresponds to the year of the GFC layer used to generate the forest loss polygon). We chose 20% cloud cover as the maximum threshold, which was derived by trial and error through visual examination of the obtained images. We downloaded the red, green, and blue bands and clipped the image to obtain a 332 × 332 pixels RGB image, which corresponds to a 5 km by 5 km area^5^. We selected only the RGB bands to directly compare with the NICFI PlanetScope data, which does not provide shortwave infrared or thermal infrared bands like Landsat-8. The code used to download images, based on the Google Earth Engine Python API, allows for flexibility in the selection of other bands and additional sensors or collections and could be used to download other types of images. For NICFI PlanetScope data, we selected the first high (4.77 m) resolution RGB monthly composite available starting from the year following the GFC-defined forest loss event. We downloaded a clipped image with a 332 × 332 pixels dimension, which corresponds to a 1.6 km by 1.6 km area.

**Fig. 2:**
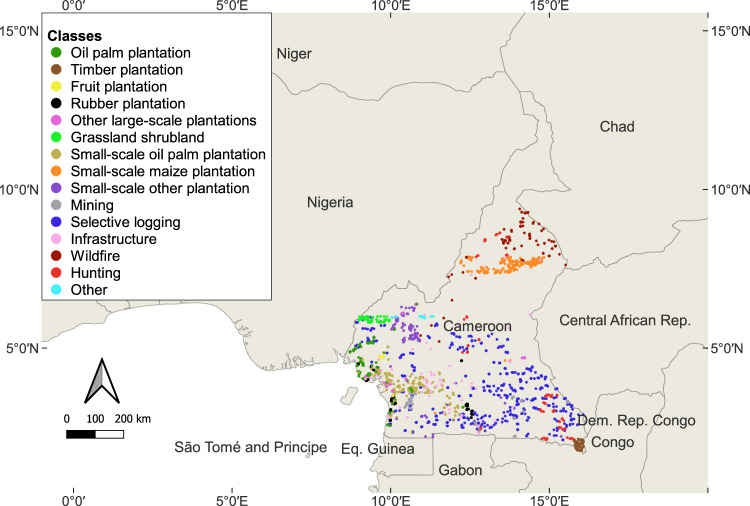
Locations of the centroids of the labelled NICFI PlanetScope images in Cameroon. The Landsat-8 dataset is similar, with around 300 fewer points, but a similar data distribution. Most data points are located in the South of the country as it is the area where most of the forest is located.

In order to allow the user the flexibility to use a time series approach to classify direct deforestation drivers, we present up to five images for each location. For Landsat-8, we selected the five images with the lowest cloud cover percentages in the five years following the forest loss event, retaining a minimum two-month time difference between each image. We kept 20% as the maximum cloud cover, which meant that not all locations provided us with five images to test our approach. In total, 84% of locations provided us with five images, and 90% with at least four images. We removed all locations that only gave us one image. For NICFI PlanetScope, we selected the first five monthly composites available starting from the year following the forest loss event, and again we also made sure to have at least two months between each composite Fig. 2.

### Auxiliary data

For each image, we added auxiliary biophysical and infrastructure information^5^, to enhance and potentially improve the performance of downstream classification tasks by the user. For all variables, we assume that the value in one pixel of the lower resolution data (i.e. auxiliary data) is the same in all the pixels of the higher resolution data (i.e. image) that cover the same area:

- Forest gain (bitmask) from GFC^27^: 30-m resolution, data for the period 2000–2012, downloaded via the Google Earth Engine Python API (although here we note that these data have not been updated since 2012, and so other products such as Tropical Moist Forest (TMF)^24^ providing forest regrowth data for 1990–2022 could provide more recent information).
- Near infrared, shortwave infrared 1 and 2 bands from Landsat-8 TOA: 30-m resolution, data every 16 days for 2013–2023, downloaded via the Google Earth Engine Python API and selected using the same process as for Landsat-8 RGB images.
- From National Centers for Environmental Prediction (NCEP) Climate Forecast System Version 2 (CFSv2) 6-hourly Products^39,40^ (https://rda.ucar.edu/datasets/ds094.0/#): surface level albedo and volumetric soil moisture content (depths: 0.1 m, 0.4 m, 1.0 m, 2.0 m) in 0.01%; radiative fluxes (clear-sky longwave flux downward and upward, clear-sky solar flux downward and upward, direct evaporation from bare soil, longwave and shortwave radiation flux downward and upward, latent, ground and sensible heat net flux), potential evaporation rate, and sublimation in W/m²; humidity (specific, maximum specific, minimum specific) in 10-4 kg/kg; ground level precipitation in 0.1 mm; air pressure at surface level in 10 Pa; wind level (u and v component) in 0.01 m/s, water runoff at surface level in 232.01 kg/ m²; temperature in K: 22264-m resolution, available four times a day for 2011-2023, downloaded directly from the NOAA website and selected the mean of the monthly mean over five years before the forest loss event, the monthly maximum over five years before the forest loss event, and the monthly minimum over five years before the forest loss event for each parameter.
- Closest street and closest city from OpenStreetMap^41^ in km: directly downloaded with the Nominatim API.
- Altitude in m, slope and aspect in 0.01° from the Shuttle Radar Topography Mission (SRTM)^42^: 30-m resolution, measured for 2000, downloaded via the Google Earth Engine API.
- Presence of peat (bitmask) from GFW^43^ (https://data.globalforestwatch.org/datasets/gfw::global-peatlands/about): 232-m resolution, measured for 2017, directly downloaded on the GFW website.

## Data Records

The dataset described in this paper is available from Zenodo^12^: https://zenodo.org/records/8325259. It is divided into different folders, following the classification scheme used, and the labels for the images are in a separate folder:

- ‘my_examples_landsat_final_detailed.zip’ contains Landsat-8 images, auxiliary data and forest loss pickle files that can be used to train, validate and test a model for the detailed classification of deforestation drivers (15 classes, Table 1).
- ‘my_examples_planet_final_detailed.zip’ contains NICFI PlanetScope images, auxiliary data and forest loss pickle files that can be used to train, validate and test a model for the detailed classification of deforestation drivers (15 classes, Table 1).
- ‘my_examples_landsat_final.zip’ contains Landsat-8 images, auxiliary data and forest loss pickle files that can be used to train, validate and test a model for the classification of deforestation drivers by groups (4 classes, Table 1).
- ‘my_examples_planet_final.zip’ contains NICFI PlanetScope images, auxiliary data and forest loss pickle files that can be used to train, validate and test a model for the classification of deforestation drivers by groups (4 classes, Table 1).
- ‘my_examples_landsat_detailed_timeseries.zip’ contains Landsat-8 images, auxiliary data and forest loss pickle files that can be used to test a model for the detailed classification of deforestation drivers (15 classes, Table 1) using multiple images and a time series analysis.
- ‘my_examples_planet_detailed_timeseries.zip’ contains NICFI PlanetScope images, auxiliary data and forest loss pickle files that can be used to test a model for the detailed classification of deforestation drivers (15 classes, Table 1) using multiple images and a time series analysis.
- ‘labels.zip’ which includes, in csv files, the labels for each image in each folder described above (image identified by folder and coordinates or ‘path’) and matches the format of the csv files used as inputs to train, validate and test our classification model. The csv files are generated when populating the ‘my_examples_XX’ folders above, based on the name of the data sources overlaid with GFC shapefiles. Each ‘all.csv’ contains all images with their labels for each sensor and classification scheme.

For ‘labels.zip’, we have subfolders for Landsat and PlanetScope. Then, for each type of imagery, we have subfolders for ‘detailed’, ‘groups’ and ‘time series’ which correspond to the different ‘my_examples’ folders listed above.

**Fig. 3:**
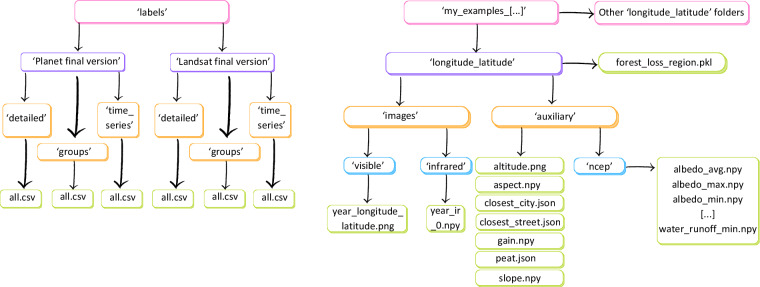
Structure of the data records for the images in our dataset. Each colour represents a folder sub-level. For instance, the final files are in green and the parent folders are in pink.

For each folder, subfolders named with the coordinates of the centre of the images contain each:

- A folder ‘images’, with a sub-folder ‘visible’ containing the PNG RGB image; and a sub-folder ‘infrared’ containing the infrared bands in a NPY file.
- A folder ‘auxiliary’ with topographic and forest gain information in a NPY format, OpenStreetMap and peat data in a JSON format, and a sub-folder ‘ncep’ containing all data from NCEP in a NPY format.
- The forest loss pickle file delimiting the area of forest loss Fig. 3.

## Technical Validation

We rely on the technical validation performed for each data source by the original data creators used to identify land use and forest loss patches. GFW^29^ data checks rely on the collaboration with the Cameroon Ministry of Forestry and Wildlife and WRI. Land Matrix^30^ uses an error-checking process involving cross checks with multiple data sources. WorldCover^31^ data has been independently validated by Wageningen University and the International Institute for Applied Systems Analysis (IIASA)^44^. Geo-Wiki^32^ data quality checks involved using control locations to produce quality scores for participants labelling drivers of forest loss and the technical validation involved a comparison with the Curtis *et al*.^45^ drivers of forest loss layer in South America. Doungous *et al*.^34^ data are individual field surveys. BIOPAMA data^36^ was validated with more than 10,000 randomly distributed reference points^35^ and existing oil palm maps^46–49^. WorldCereal^50^ relies on high quality reference datasets provided by the agricultural community and accuracy checks by users. GlobFire data^37^ was validated by comparison with other wildfires databases^51–57^.

We still performed additional steps to make sure of the quality of our dataset:

- We cross-checked the data when the same information was provided through multiple data sources, and removed land-use shapefiles where the detailed land uses did not match or kept only one shapefile when there were duplicates.
- We removed all land use shapefiles where the detailed land use was uncertain, even if auxiliary sources such as company websites suggested specific land uses in certain locations.
- We determined the 20% threshold for cloud cover by trial and error to find a balance between having enough images and having images satisfactory enough (i.e. not too cloudy) to be able to classify drivers. The quality of the images was determined by visual examination of the results with different thresholds.
- We cleaned up our image dataset to remove downloaded satellite images with a size below 10kB, which corresponded to blank images and not the RGB images expected.

## Usage Notes

This data can be used fully or partially to train, validate and test the classification of direct deforestation drivers or follow-up land use after deforestation. The code provided can also be re-purposed for other locations, or other types of imagery.

All data that has been used, modified and re-distributed is compliant with the data sharing licenses. The NICFI PlanetScope images fall under the same license as the NICFI data program license agreement. OpenStreetMap® is open data, licensed under the Open Data Commons Open Database License (ODbL). The rest of the data is under a Creative Commons Attribution 4.0 International License.

## Data Availability

The code used to prepare data is available on Github, in the ‘prepare_files’ folder and the code to format the folders in ‘model’ > ’data’ > ’ForestNetDataset’: https://github.com/aedebus/Cam-ForestNet. The folders are organised to be ready-to-use with our classification model, Cam-ForestNet, by simply unzipping the relevant ‘my_examples’ folder in ‘model’ > ’data’ > ’ForestNetDataset’. QGIS 3.24.0, Python 3.8./3.5., and the Google Earth Engine Python API were used. Ubuntu 20.04.3 LTS was used to run the code.
